# Anxiety and Depression Among Non-Facial Burn Patients at a Tertiary Care Center in Pakistan

**DOI:** 10.7759/cureus.11347

**Published:** 2020-11-05

**Authors:** Dujanah S Bhatti, Nur Ul Ain, Reesham Zulkiffal, Zuhdi Sufian Al-Nabulsi, Ahmad Faraz, Raheel Ahmad

**Affiliations:** 1 Plastic Surgery, Aberdeen Royal Infirmary, Aberdeen, GBR; 2 Plastic and Reconstructive Surgery, Holy Family Hospital, Rawalpindi, PAK; 3 Anatomy, Foundation University Medical College, Rawalpindi, PAK; 4 Urology, Belford Hospital, Fort William, GBR; 5 Trauma and Orthopaedics, Leeds Teaching Hospitals NHS Trust, Leeds, GBR; 6 Surgery, Holy Family Hospital, Rawalpindi, PAK

**Keywords:** burn, clinical anxiety, depression

## Abstract

Introduction: A patient who suffers from burn injuries can be subjected to various mental and psychological conditions that can adversely affect their health and wellbeing.

Material and methods: A cross-sectional study was conducted between 1st September 2019 and 30th March 2020 in a tertiary care hospital in Pakistan. Patients were selected in the outpatient department and follow-up was done at two and four weeks following definitive. Some 225 patients in our study fulfilled the inclusion criteria. Patients were assessed using Urdu translated scales. Hamilton Anxiety Rating Scale (HAM-A) and Hamilton Depression Rating Scale (HAM-D) were used. Data were analyzed with the help of SSPS software version 13.0.

Result: Out of 119 (52.8 %) male patients, the highest percentage was of accidental injuries 106 (89%) followed by suicidal burns 9 (7.5%). A similar trend was seen in females; out of n=106 females, 92 (86%) presented with accidental burn injuries and only 11 (10%) patients have a history of suicidal burns. A fraction of the sample had a history of homicidal burn injuries, with 4 (3%) male and 3 (2%) female patients.

The variation of anxiety level and depth of burn varied considerably. Among patients who suffered superficial thickness burns (n=105, 47%), 69.5% of patients experienced mild anxiety symptoms. Only 28 (26.6%) patients had moderate anxiety and severe anxiety was the lowest, at only 3.8% (n=4). A similar trend was observed in deep burn patients, but the level of severe anxiety was significantly higher at 26%. This was statistically significant (p < 0.05).

Deep burn patients had the highest percentage (n=54, 45.3%) of very severe depression compared to only 10% in superficial burns. The variation between the two categories was statistically significant (p < 0.05). The majority of (35.2%) patients experienced mild symptoms of depression and this correlated with superficial burn injuries.

Conclusion: A burn injury can seriously affect the mental wellbeing of patients. With the severity of burn injury we saw that severe depression was prevalent. This aspect must be taken into consideration when treating such patients and it warrants a multidisciplinary team (MDT) strategy.

## Introduction

A patient who suffers from burn injuries can be subjected to various mental and psychological conditions that can adversely affect their health and wellbeing. Understanding the nature of their ailment and the impact it has on their health involves a detailed study of the nature of burn, socioeconomic factors, personal life, and reason for the injury [1]. These terrible life events cause extreme stress in these patients [2].

Application of heat, electric current, inflammable material, chemical to the internal or external surface of the body can cause a burn [3]. These agents can significantly damage the surface in contact. The resulting injury can be formed as a superficial burn to deep burn [4]. The distress caused by such injuries can have a disastrous impact on the physical as well as psychological health of patients [5]. Stress, anxiety, depression, low self-esteem, body disfigurement, social isolation, unemployment, financial burden, family problems, etc. are faced by these patients. Thus, having a holistic treatment approach is mandatory for completed recovery [6].

Burn-related pain depends on various factors. These included the size, depth, total body surface area, mode of injury, timing of the injury, general state of the patient, comorbidities, and general pain tolerance of the patients. It has been documented by Byers et al. that the pain threshold of patients with burn injuries was significantly higher when compared to patients with other injuries. Therefore, minor procedures like a change of dressing became very difficult [7]. This is clearly demonstrated by Woo in his work, which concluded that there is a strong association between pain and anxiety and this relation also extends into the aspects of depression [8]. Studies have elucidated that this procedure-induced pain is progressive as the treatment continues [9].

Worldwide, burns are the fourth leading cause of trauma after motor vehicle collision followed by falls and violence. This accounts for 5%-12% of all injuries worldwide and these 11.5 million patients require some sort of medical attention [10]. In our region, the annual incidence of burn injuries in the Eastern Mediterranean and South-East Asia regions is estimated to be 187 and 243 per 100,000 population, respectively. In Pakistan the influx of patients in an emergency is 147 per 100,000 population, this was concluded in a study conducted by Siddiqui et al. [10].

The complications of burn are various but the most seen in our setup are scars and contractures. These cause visible deformity and if present on the face, head, or neck can also contribute to social anxiety. Although sex difference is not accounted for when calculating the morbidity and mortality of burn injuries. Historically, there is increasing evidence that women sustain higher morbidity due to burning injuries. These can also result in depression, and women are more susceptible to this. This vulnerable group of individuals requires more detailed care and a multidisciplinary team (MDT) approach to restore their quality of life and functional capacity as close to normal as possible.

## Materials and methods

We designed a cross-sectional study, conducted between 1st September 2019 and 30th March 2020 in a tertiary care hospital in Pakistan. Patients were enrolled from the Burn and Reconstructive department of the hospital for our study based on a defined inclusion and exclusion criteria. Patients were selected in the outpatient department and follow-up was done at two and four weeks following definitive treatment. Ethical consideration was undertaken by the Ethical Board of the hospital and was approved. We enrolled 225 patients based on the following criteria.

All patients who presented in the Burn unit outpatient clinic during the time the study was undertaken. The age bracket was from 18 to 80 years. Informed consent was taken from the patient before enrolling them in the study. Patients were excluded if they had an amputated body part or another psychotic ailment that precluded their judgment. Facial burns were also excluded from the study. Data were collected on a pre-designed questionnaire that was filled by the patient. This was designed in the local language for easy understanding.

Patients were assessed using Urdu translated scales. Hamilton Anxiety Rating Scale (HAM-A) and Hamilton Depression Rating Scale (HAM-D) were used. Each scale consists of a set number of questions with multiple-choice options. Each option is graded from 0 to 4 based on the severity of the symptom. The cumulative score of these scales is judged and graded from mild to severe. Clinical and demographic variables were also studied. These included gender and area of the burn.

All patients were interviewed to exclude pre-existing psychiatric ailment. Mode of injury was studied along with social and homicidal attempts.

Data were analyzed with the help of SSPS software version 13.0 using basic descriptive statistics such as mean, standard deviation, and median. The Chi-square test was used where appropriate. Statistically significant value was set at a p-value of less than 0.05.

## Results

We enrolled 225 patients in our study who fulfilled the inclusion criteria. Male patients were slightly more than females. Out of 119 male patients, the highest percentage was of accidental injuries (106) followed by suicidal burns (n=9). A similar trend was seen in females, where out of n=106 females, 92 presented with accidental burn injuries and only 11 patients has a history of suicidal burns. A fraction of the sample had a history of homicidal burn injuries, with four male and three female patients (Table 1).

**Table 1:** Clinical and demographic aspects of burn patients.

Variable	Male	Female	Total
Accidental	106 (89%)	92 (86%)	225
Homicidal	4 (3%)	3 (2%)	
Suicidal	9 (7.5%)	11 (10%)	
	n=119	n=106	

While analyzing the level of anxiety in our sample, it was found that the patients experienced anxiety symptoms from mild to severe. However, the variation of anxiety level and depth of burn varied considerably. In the subject presented with superficial burns (n=105, 47%), 69.5% of patients had mild anxiety. Only 28 (26.6%) patients had moderate anxiety and severe anxiety was the lowest, at only 3.8% (n=4). A similar trend was observed in deep burn patients, but the level of severe anxiety was significantly higher at 26%. This was statistically significant (p < 0.05).

Depression was observed in all individuals in all grades. The majority (n=37) of patients experience mild symptoms. These were patients with superficial burn injuries. An almost similar number of patients in this category had moderate and severe depressive symptoms. Deep burn patients had the highest percentage (n=54, 45.3%) of very severe depression, followed by severe depression at 40%. The variation between the two categories was statistically significant (p < 0.05) (Table 2).

**Table 2:** Anxiety and depression with various factors of burn patients.

Anxiety	Superficial burn n=105 (47%)	Deep burn n=119 (53%)	
Mild	73 (69.5%)	61 (51.2%)	The result is significant at p < 0.05
Moderate	28(26.6%)	27 (22.6%)	
Severe	4 (3.8%)	31 (26%)	
Depression			
Mild	37 (35.2%)	14 (10.9%)	The result is significant at p < 0.05
Moderate	28 (26.6%)	11 (9.2%)	
Severe	29 (27.6%)	40 (33.6%)	
Very severe	11 (10.4%)	54 (45.3%)	

## Discussion

Burn can have a significant impact on a person’s psychological and emotional wellbeing [11]. Despite the trauma of burn injury, most patients adapt well to post-burn life. Initially, some difficulties are there adjusting to the trauma but it gets better with time. However, sometimes the symptoms are prolonged and worsen. It is extremely difficult for burn survivors to live with noticeable scars in a society that values physical appeal. Disfigurement can lead to body image dissatisfaction which can in turn cause social anxiety, social withdrawal, and depression [12]. Such psychological response has been observed even in the previously well-adjusted patients [13]. Other contributing factors related to the stress of burn injury are functional limitations, long and demanding rehabilitation programs, secondary complications, previous history of emotional disturbances, etc. In our study, we investigated the association of anxiety and depression with burn injury characteristics in non-facial burns. The results of this study show that symptoms of anxiety and depression are related to the depth of burn injury.

We observed that most of the patients with superficial burns suffered from mild anxiety and while patients with deeper burn tend to fall victim to severe anxiety. This trend was also observed by Dahl [12]. In our study, patients with deep burn tended to suffer from severe anxiety and depression which is corroborated by some studies [14-15]. This prevalence can be due to more morbidity associated with the deep burn. Moreover, deep burn injury results in more conspicuous scars which are not only disfiguring but also functionally limiting and rehabilitation can be demanding and prolonged compared to superficial burns. We realized the importance of psychosocial support for patients treated for burn victims to help them cope and adapt to burn injury [16-17]. A psychologist must be an integral part of the burn care team to help address psychological issues related to the burn trauma. The patient must be made aware of their emotional responses, the psychological impact, and how to deal with it [18]. In addition to the patient, their family should also be involved in the process of holistic healing and for that nurses can play a vital role [19]. A special consideration is to identify high-risk patients, i.e. patients with extensive and deep burns, early on in the treatment as they are more prone to severe anxiety and depression.

## Conclusions

Our study found that all non-facial burn victims appeared to be affected by anxiety and depression symptoms ranging from mild to severe post sustaining their burn injuries. It suggests that those symptoms are related to the degree of burns where severe anxiety and depression will be noticed more with deep burns rather than superficial. Hence, we advise a formal MDT approach for mainly treating patients with deep burns. We suggest running larger multi-center studies looking into the prevalence of anxiety and depression in patients of burn injuries considering longer follow-up schemes. 
